# Supplementary material for: Cohesin and CTCF control the dynamics of chromosome folding
Source: Nat Genet. 2022 Dec 5;54(12):1907–18. doi: 10.1038/s41588-022-01232-7 (PMC9729113; doi:10.1038/s41588-022-01232-7)
Supplement: Supplementary file 1 — Supplementary methods, polymer modeling, Fig. 1, descriptions for Videos 1–3 and Tables 1–4, flow cytometry gating strategy and references. [file 41588_2022_1232_MOESM1_ESM.pdf]

nature genetics

Article

https://doi.org/10.1038/s41588-022-01232-7

# Cohesin and CTCF control the dynamics of chromosome folding

In the format provided by the authors and unedited

# Table of Contents


**1 Methods** **3**

1.1 Generation of targeting vectors for random integration of TetO array and TetR-tdTomato . . . . 3
1.2 Generation of targeting vectors for TetO and LacO . . . . . . . . . . . . . . . . . . . . . . . . . . . 3
1.3 Removal of CTCF sites by Cre recombination . . . . . . . . . . . . . . . . . . . . . . . . . . . . . 4
1.4 Generation of control cell lines expressing OsTir1 . . . . . . . . . . . . . . . . . . . . . . . . . . . 4
1.5 Tagging of endogenous *Rad21* lcous and removal of resistance genes *NeoR* and *HygroR* . . . 4
1.6 Removal of CTCF sites in dual array TetO-LacO cell lines . . . . . . . . . . . . . . . . . . . . . 5
1.7 Generation of control cell lines to measure localization error and experimental uncertainty . . . 5
1.8 piggyBac insertion site mapping . . . . . . . . . . . . . . . . . . . . . . . . . . . . . . . . . . . . 5
1.9 Capture-Hi-C sample preparation . . . . . . . . . . . . . . . . . . . . . . . . . . . . . . . . . . . 6
1.10 Targeted nanopore sequencing with Cas9-guided adapter ligation (nCATS) . . . . . . . . . . . 6
1.11 Hi-C sample preparation . . . . . . . . . . . . . . . . . . . . . . . . . . . . . . . . . . . . . . . . 6
1.12 4C-Seq sample preparation . . . . . . . . . . . . . . . . . . . . . . . . . . . . . . . . . . . . . . 7
1.13 Cell cycle analysis of RAD21 depleted cells by flow cytometry . . . . . . . . . . . . . . . . . . . 7
1.14 Western Blot . . . . . . . . . . . . . . . . . . . . . . . . . . . . . . . . . . . . . . . . . . . . . . . 7
1.15 Estimation of sister chromatids . . . . . . . . . . . . . . . . . . . . . . . . . . . . . . . . . . . . 8
1.16 Median convergent CTCF distance . . . . . . . . . . . . . . . . . . . . . . . . . . . . . . . . . . 8
1.17 Analysis of piggyBac insertion site mapping . . . . . . . . . . . . . . . . . . . . . . . . . . . . . 8
1.18 Hi-C analysis . . . . . . . . . . . . . . . . . . . . . . . . . . . . . . . . . . . . . . . . . . . . . . . 9
1.19 Loop analysis . . . . . . . . . . . . . . . . . . . . . . . . . . . . . . . . . . . . . . . . . . . . . . 9
1.20 4C-Seq analysis . . . . . . . . . . . . . . . . . . . . . . . . . . . . . . . . . . . . . . . . . . . . . 9
1.21 Nanopore sequencing analysis . . . . . . . . . . . . . . . . . . . . . . . . . . . . . . . . . . . . . 9
1.22 Capture-Hi-C analysis . . . . . . . . . . . . . . . . . . . . . . . . . . . . . . . . . . . . . . . . . 9
1.23 Differential Capture-Hi-C maps . . . . . . . . . . . . . . . . . . . . . . . . . . . . . . . . . . . . 9
1.24 Conversion of simulation steps to real time . . . . . . . . . . . . . . . . . . . . . . . . . . . . . 10
1.25 Metrics for quantifying similarity between simulations and experiments . . . . . . . . . . . . 10

**2 Polymer modelling** **11**

2.1 Introduction . . . . . . . . . . . . . . . . . . . . . . . . . . . . . . . . . . . . . . . . . . . . . . . 11
2.2 Subdiffusion of the difference vector in the Rouse chain . . . . . . . . . . . . . . . . . . . . . . 11
2.3 Subdiffusion of the distance . . . . . . . . . . . . . . . . . . . . . . . . . . . . . . . . . . . . . . 13
2.4 Bead-spring chain with excluded volume . . . . . . . . . . . . . . . . . . . . . . . . . . . . . . . 14

**3 Supplementary Figures** **16**

3.1 Supplementary Figure 1: Features of HMM states called on simulations across the parameter space . . . . . . . . . . . . . . . . . . . . . . . . . . . . . . . . . . . . . . . . . . . . . . . . . 16

**4 Supplementary Videos** **17**

4.1 Supplementary Video S1: Live-cell imaging of TetO arrays upon depletion of RAD21 . . . . . . 17
4.2 Supplementary Video S2: Dynamics of LacO-TetO radial distances . . . . . . . . . . . . . . . 17
4.3 Supplementary Video S3: Cohesin and CTCF decrease average LacO-TetO radial distances . 17

**5 Supplementary Tables** **18**

5.1 Supplementary Table S1: Statistics of live-cell imaging data . . . . . . . . . . . . . . . . . . . 18
5.2 Supplementary Table S2: Statistical tests for duration and frequency of the HMM-called states 18
5.3 Supplementary Table S3: Oligonucleotides . . . . . . . . . . . . . . . . . . . . . . . . . . . . . 18

5.4 Supplementary Table S4: Spot detection and tracking parameters . . . . . . . . . . . . . . . . . 18

**6 Flow cytometry gating strategy** **19**

**References** **20**

# 1 Methods

## 1.1 Generation of targeting vectors for random integration of TetO array and TetR-tdTomato

To generate an 8 kb TetO array within piggyBac ITRs (inverted terminal repeats), the TetO array was obtained from the pSO2.Pac.TetO, a gift from Edith's Heard lab,[1] by growing bacteria at 37 °C to reduce the size of the original 30 kb operator array by recombining. The array was then excised from the vector by restriction digest with BamHI (NEB, R0136S) and cloned into the PB-empty[2] (final vector PB-empty-DSE-TetO-8kb). A cassette carrying three strong CTCF sites was excised with XhoI (NEB, R0146S) from PB-empty_DSE_TetO_2.7kb_3xCTCF[2]) and ligated into the PB-empty-DSE-TetO-8kb vector using T4 DNA Ligase (NEB, M0202L). Clones were screened by Sanger sequencing (Microsynth) for CTCF sites inserted facing toward the TetO array. The final vector (PB-3xCTCF-TetO) was validated by restriction digest with EcoRI (NEB, R3101L) and NotI (NEB, R3189L) for the correct size of the operator array and Sanger sequencing (Microsynth) for the correct insertion of the CTCF cassette.

To express the Tet repressor (TetR) and Lac repressor (LacI) fused to a fluorescent protein flanked by ITRs for piggyBac transposition, the PB-empty vector was first linearized by digestion with XhoI (NEB, R0146L) and the TetR-eGFP was amplified with Phusion High-Fidelity DNA Polymerase (Thermo Fisher Scientific, F530L) from pBroad3-TetR-ICP22-EGFP kindly provided by Tim Pollex[3] with Gibson overhangs. PB-Ubc-TetR-eGFP was assembled using Gibson cloning (NEB, E2611L). The tdTomato was amplified with Gibson overhangs and assembled with the digested PB-TetR-eGFP (BamHI, NEB, R3136L and EcoRI, NEB, R3101L) to yield the PB-Ubc-TetR-tdTomato vector. To increase expression levels of the fusion proteins, the Ubc promoter was exchanged for the stronger CAGGS promoter that was amplified with Gibson overhangs from a pCAGGS plasmid. The final PB-TetR-tdTomato was made using Gibson assembly of the amplified CAGGS promoter (from Addgene plasmid #20733) with the PB-Ubc-TetR-tdTomato digested with BglII (NEB, R0144L) and AgeI (NEB, R3552S). To generate PB-CAGGS-TetR-eGFP, PB-Ubc-TetR-eGFP and PB-TetR-tdTomato were digested with XhoI (NEB, R0146L) and ligated using T4 DNA Ligase (NEB, M0202L). PB-LacI-eGFP was generated by amplification of the LacI with overhangs for subsequent Gibson assembly with the digested PB-TetR-eGFP (AgeI, NEBR3552S). Primers used for cloning can be found in **Suppl. Table S3**.

## 1.2 Generation of targeting vectors for TetO and LacO

Vector for targeting the TetO array to the genomic locus on chr15:11,647,372: The vector pMK-chr15-Rox-PuroR-Rox containing the homology arms for chromosome 15 as well as the Puromycin resistance gene flanked by Rox sites was custom synthesized by GeneArt Synthesis (Thermo Fisher Scientific) and linearized with SbfI (NEB, R0642S) and SpeI (NEB, R3133S). A short linker sequence including a XhoI restriction site was introduced into the vector by PCR amplification from pMK-chr15-Rox-PuroR-Rox with Gibson overhangs. The XhoI restriction site was then used to ligate the 3xCTCF-TetO (cut from the PB-3xCTCF-TetO vector) into the vector leading to pMK-3xCTCF-TetO-Rox-PuroR-Rox. Vector for targeting the LacO array to the genomic locus on chr15:11,496,908: The vector pUC19-empty was linearized with EcoRI (NEB, R3101L) and BamHI (NEB, R3136L). The left homology arm was amplified from E14 wild-type genomic DNA with overhangs for Gibson assembly. The 5'-ITR was amplified from PB-empty with Gibson overhangs and both PCR products were assembled into the pUC19 vector (Addgene, #50005) to yield pUC19-lHA-5'ITR. pUC19 was digested with KpnI (NEB, R3142L) and BamHI (NEB, R2126L) to be assembled into pUC19-3'ITR-3xCTCF-rHA with the following PCR products with respective Gibson overhangs: The 3'ITR was amplified from PB-empty, the CTCF cassette was amplified from pMK-chr15-Rox-PuroR-Rox and the right homology arm was amplified from E14 wild-type genomic DNA. To make the targeting vector, pUC19-lHA-5'ITR was linearized with EcoRI and BamHI,

pUC19-3'ITR-3xCTCF-rHA was linearized with KpnI and HindIII (NEB, R3104L) and the Neomycin resistance gene was amplified from pEN113 (Addgene, #86233). All three parts were assembled using Gibson assembly to pUC19-ITR-NeoR-ITR-3xCTCF. The LacO array was excised from the pLAU43_LacO_plus vector[4] with XhoI (NEB, R0146L) and ligated into the linearized targeting vector (cut with XhoI) using T4 DNA Ligase resulting in the final targeting vector pUC19-ITR-NeoR-ITR-3xCTCF-LacO. Primers used for cloning can be found in **Suppl. Table S3**.

## 1.3 Removal of CTCF sites by Cre recombination

To selectively remove the three CTCF binding sites flanking the operator arrays, $0.5 \times 10^6$ cells of each cell line were transfected 1 µg of pIC-Cre (a gift from the Schübeler lab) using Lipofectamine3000 according to the manufacturer's instructions (Thermo Fisher Scientific, L3000008). 7 days after transfection, the cells were sorted and genotyped as described previously. Primers used in genotyping are listed in **Suppl. Table S3**.

## 1.4 Generation of control cell lines expressing OsTir1

E14 wild-type cells were transfected with the targeting vector pEN396-pCAGGS-Tir1-V5-2A-PuroR TIGRE donor and the gRNA vector pX330-EN1201 (Addgene #92144 and #92142) using nucleofection with the Amaxa 4D-Nucleofector X-Unit and the P3 Primary Cell 4D-Nucleofector X Kit (Lonza, V4XP-3024 KT). $2 \times 10^6$ cells were harvested using accutase (Sigma Aldrich, A6964) and resuspended in 100 µl transfection solution (82 µl primary solution, 18 µl supplement, 15 µg Tir1 targeting vector and 5 µg of pX330-EN1201) and transferred to a single Nucleocuvette (Lonza). Nucleofection was performed using the protocol CG110. Transfected cells were directly seeded in pre-warmed E14 standard medium. 48 hours after transfection, 1 µg/ml of puromycin (InvivoGen, ant-pr-1) was added to the medium for 3 days to select cells for insertion of the Tir1 integration. Cells were sorted and genotyped as described previously. Primers used in genotyping are listed in **Suppl. Table S3**.

## 1.5 Tagging of endogenous *Rad21* lcous and removal of resistance genes *NeoR* and *HygroR*

The gRNA vector and the targeting vector from Liu et al.[5] were purchased from Addgene (gRNA pX330-EN1082: #156450, targeting vector pEN313: #156431). The gRNA sequence can be found in **Suppl. Table S3**. The targeting vector was modified using a restriction digest with NdeI and EcoRI (NEB, R0111S and NEB, R3101L) and subsequent Gibson assembly (NEB, E2611L) to insert an FKBP-tag at the end of the coding sequence as well as a Rox-HygroR-Rox cassette for selection (final vector: RAD21-Halo-FKBP-Rox-HygroR-Rox). The clonal lines carrying the TetO and LacO cassettes as well as TeR-tdTomato and LacI-eGFP (clones 1B4 and 2C10) were transfected with the targeting vector (RAD21-Halo-FKBP-Rox-HygroR-Rox) and the gRNA vector PX330-EN1082 using nucleofection with the Amaxa 4D-Nucleofector X-Unit and the P3 Primary Cell 4D-Nucleofector X Kit (Lonza, V4XP-3024 KT) as described above. 48 hours after transfection, 160 µg/ml of Hygromycin B (Thermo Fisher Scientific, 10687010) was added to the medium for 3 days to select cells for insertion of the HaloTag-FKBP-tag cassette. 7 days after selection, $0.5 \times 10^6$ cells were transfected with 2 µg of pCAGGS-Dre-IRES-bsd (purchased from Gene Bridges) using Lipofectamine3000 according to the manufacturer's instructions (Thermo Fisher Scientific, L3000008) to remove the Neomycin and Hygromycin resistances used as selection markers for previous integrations. Prior to single-cell sorting, the cells were incubated with 100 nM JF646 HaloTag Ligand[6] in full culturing medium for 30 min at 37 °C, 8% $CO_2$ and washed three times with PBS. The cells were sorted for fluorescent emission at 664 nm. Sorted cells were cultured and genotyped as described for the Tir1 integration. Primers used for genotyping are listed in **Suppl. Table S3**.

Cell lines showing the corrected genotype were selected, expanded and validated for homozygous insertion of the HaloTag-FKBP-tag and correct functioning of the degron system by Western Blot (as described below). Clones 1B1 (-PuroR,-NeoR,-HygroR) and 2C11 (+PuroR,+NeoR,-HygroR) were used for further engineering.

## 1.6 Removal of CTCF sites in dual array TetO-LacO cell lines

To selectively remove the three CTCF binding sites flanking the operator arrays, 0.5x10$^6$ cells of the TetO-LacO dual array cell line + RAD21-HaloTag-FKBP (clone 1B1) were transfected 1 µg of pIC-Cre using Lipofectamine3000 according to the manufacturer's instructions (Thermo Fisher Scientific, L3000008). 5 days after transfection, 0.5x10$^6$ cells of the pool were further transfected with 1 µg pCAG-FlpO-P2A-HygroR.[7] Cells were then sorted and genotyped as described previously. Primers used for genotyping are listed in **Suppl. Table S3**. Since the recombination with Flippase did not work sufficiently to attain correct clones, the CTCF sites flanking the LacO array were then removed using CRISPR-Cas9 deletion. The gRNA sequences for the CRISPR/Cas9 knock-out of the CTCF sites flanking the LacO array were designed using the online tool `https://eu.idtdna.com/site/order/designtool/index/CRISPR_SEQUENCE` and purchased from Microsynth. The gRNA sequence can be found in **Suppl. Table S3**. The gRNA sequence was cloned into the PX330 plasmid (Addgene, #58778) using the BsaI restriction site as described previously. The pool of cells transfected with pIC-Cre was further transfected with 0.5 µg of each gRNA/Cas9 vector and subsequently sorted and genotyped. Correct clones were expanded and validated by genotyping PCR that was analyzed on a 1% agarose gel imaged with a Typhoon FLA 9500 scanner (GE Healthcare). Subsequent Sanger sequencing of the PCR product (Microsynth) confirmed the removal and clones 1B1 (+CTCF sites, -promoters, +RAD21-HaloTag-FKBP), 1A2 (-CTCF sites, -promoters, , +RAD21-HaloTag-FKBP) and 1F4 (-CTCF sites, +promoters from resistance gene cassette, +RAD21-HaloTag-FKBP) were used in live-cell imaging and Capture-Hi-C experiments.

## 1.7 Generation of control cell lines to measure localization error and experimental uncertainty

To generate a control cell line for the dual array imaging, one clone of RAD21-AID-eGFP + 3xCTCF-TetO + TetR-tdTomato (clone 2B10) was transfected with 200 ng pBroad3_hyPBase_IRES_tagRFPt and 200 ng PB-TetR-eGFP using Lipofectamine3000 (Thermo Fisher Scientific, L3000008) according to the manufacturer's instructions. Cells were cultured in standard E14 medium for 7 days and sorted (as described previously) for fluorescent emission at 507 nm (eGFP) and 581 nm (tdTomato). Clonal lines were screened for a good SNR by microscopy on Corning High-Content Imaging Glass Bottom Microplates (96-well, Corning, 4580) and were used for estimation of localization error and the experimental uncertainty on the distance by live-cell imaging using the same analysis pipeline as for the TetO-LacO dual-array cell line (see description below).

## 1.8 piggyBac insertion site mapping

The integration sites of the random integrations by PiggyBase were mapped as described in Redolfi et al.[2] In short, genomic DNA (2 µg) was fragmented to an average of 500 bp by sonication (Covaris) and ligation of full-length barcoded Illumina adapters was performed using the TruSeq DNA PCR-free kit (Illumina) according to the manufacturer's guidelines, with the exception that large DNA fragments were not removed. Libraries were pooled together and capture of desired fragments was performed using biotinylated probes against the piggyBac inverted terminal repeats (ITRs) sequences using xGEN Hybridisation reagents (IDT). Following capture, libraries were amplified for 14 cycles (KAPA HiFi Hotstart). Sequencing was performed on the NextSeq500 platform (Illumina) as paired-end 300 cycles.

## 1.9 Capture-Hi-C sample preparation

Capture-Hi-C sample preparation was performed as described previously.[7] In short, for RAD21 depletion, 2x$10^7$ cells were treated with 500 nM dTag-13 (Sigma-Aldrich, SML2601-1MG) for 2 h at 37 °C. All cells were then crosslinked with 1% formaldehyde (EMS, 15710) for 10 min at RT. The reaction was quenched with glycine (final concentration 0.125 M). Lysis was performed in 1 M Tris-HCl pH 8.0, 5 M NaCl and 10% NP40 (Sigma-Aldrich, I8896-50ML) and Complete protease inhibitor (Sigma-Aldrich, 11836170001). Cells were digested using 100 U of MboI (NEB, R0147) and ligated at 16 °C with 10,000 U of T4 DNA ligase (NEB, M0202) in ligase buffer supplemented with 0.8% Triton X-100 (Sigma-Aldrich, T8787) and 240 µg of BSA (NEB, B9000). De-crosslinking was achieved with 400 µg Proteinase K (Macherey Nagel, 740506) at 65 °C. The 3C sample was purified using a phenol/chloroform extraction. 3C library preparation and target enrichment using a custom-designed collection of 6979 biotinylated RNA "baits" targeting single MboI restriction fragments chr15:10,283,500-13,195,800 (mm9) (**Suppl. Table S3**; Agilent Technologies; as in Ref.[7]) were performed following the SureSelectXT Target Enrichment System for Illumina Paired-End Multiplexed Sequencing Library protocol. However, 9 µg of 3C input material instead of 3 µg was used for the capture and DNA was sheared using Covaris sonication with the following settings: Duty Factor: 10%; Peak Incident Power (PIP): 175; Cycles per Burst: 200; Treatment Time: 480 s; Bath Temperature: 4 °C to 8 °C).

## 1.10 Targeted nanopore sequencing with Cas9-guided adapter ligation (nCATS)

nCATS was performed as described previously in Zuin et al.[7] In short, 3-5 gRNAs sequences each (targeting the upstream and downstream regions 2-3 kb external of the respective integration cassette, either LacO or TetO integration) were designed using the IDT online tool `https://eu.idtdna.com/site/order/designtool/index/CRISPR_SEQUENCE` (**Suppl. Table S3**). Custom designed Alt-R CRISPR-Cas9 crRNAs (3-5 crRNAs targeting the region 5' and 3-5 crRNAs targeting the region 3' of the integrated transgene), Alt-R CRISPR-Cas9 tracrRNA (IDT, 1072532) and Alt-R S.p. Cas9 enzyme (IDT, 1081060) were purchased from IDT. Genomic DNA from clones 2G5 and 1F11 was extracted with Gentra Puregene Cell Kit (Qiagen, 158745) following the manufacturer's instructions. Quality of the High Molecular Weight (HMW) DNA was checked with the TapeStation (Agilent) and 5 µg of HMW DNA were de-phosphorylated using Shrimp Alkaline Phosphatase (rSAP; NEB, M0371) for 3 min at 37 °C followed by 5 min at 65 °C. To assemble the Alt-R guide RNA duplex (crRNA:tracrRNA), the six Alt-R CRISPR-Cas9 crRNAs were pooled to a final concentration of 100 µM and subsequently incubated in a ratio of 1:1 with 100 µM of Alt-R CRISPR-Cas9 tracrRNA at 95 °C for 5 min. 4 pmol of Alt-R S.p Cas9 enzyme were incubated with 8 pmol Alt-R guide RNA (crRNA:tracrRNA) at RT for 20 min to assemble the RNP complex. *In vitro* digestion and A-tailing of the DNA were performed by adding 10 µl of the RNP complex, 10 mM of dATP (NEB, N0440) and 5 U of Taq Polymerase (NEB, M0267) and incubating the samples at 30 min, 37 °C followed by 5 min, 72 °C. Nanopore sequencing adaptors were ligated using the Ligation Sequencing Kit (Oxford Nanopore Technologies, SQK-CAS109) according to the manufacturer's instructions. After purification with AMPure PB beads (Beckman Coulter, A63881), samples were loaded into MinION selecting SQL-CAS109 protocol (Oxford Nanopore Technologies).

## 1.11 Hi-C sample preparation

Hi-C sample preparation was performed as described previously in Redolfi et al.[2] Briefly, 6×$10^6$ cells were treated with 500 µM auxin (Sigma-Aldrich, I5148-2G) for 90 min and cells were crosslinked with 1% formaldehyde (EMS, 15710) and quenched with 0.125 M glycine for 5 min at RT. Cells were lysed in 10 nM Tris-HCl pH 8.0, 10 nM NaCl, 0.2% NP-40 (Sigma-Aldrich, I8896-50ML), complete protease inhibitor (Sigma-Aldrich, 11836170001) and nuclei were digested with 400 U of MboI (NEB, R0147) at 37 °C overnight. End-repair was performed using 40 µM Biotin-11-dATP (Life Technologies, 19524-016) and 50 U DNA Polymerase I

Large Klenow fragment (NEB, M0210M) incubating at 37 °C for 45 min. The end-repaired samples were ligated using 10,000 U T4 DNA ligase (NEB, M0202M) in ligase buffer supplemented with 0.8% Triton X-100 (Sigma-Aldrich, T8787) and 120 µg BSA (NEB, B9000) at 16 °C overnight. De-crosslinking was performed by adding 20 µl Proteinase K (20 mg/ml, Macherey-Nagel, 740506) to the ligation mix (1.2 ml) and incubating at 65 °C overnight. DNA was purified using phenol/chloroform and 2 µg of purified 3C sample was sonicated using the Bioruptor Pico (Diagenode). Biotinylated DNA was captured using MyOne Streptavidin T1 magnetic beads (Life Technologies, No. 65601) followed by A-tailing. Library preparation was performed according to NEBNext Ultra DNA Library prep kit instruction (NEB, E7370L) and samples were purified with magnetic AMPure bead (Beckman Coulter, A63881). Hi-C libraries were sequenced on an Illumina Nextseq500 platform (2x42 bp paired-end).

## 1.12 4C-Seq sample preparation

Sample preparation for 4C-seq was performed as previously described.[8] In short, $10^7$ cells were treated with 500 µM auxin (Sigma-Aldrich, I5148-2G) for 90 min and cross-linked in 2% paraformaldehyde (EMS, 15710) for 10 min and quenched with 0.125 M glycine (final concentration). Lysis was performed in 150 mM NaCl, 50 mM Tris-HCl (pH 7.5), 5 mM EDTA, 0.5% NP-40 (Sigma-Aldrich, I8896-50ML), 1% Triton X-100 (Sigma-Aldrich, T8787). The samples were digested with 200 U DpnII (NEB, R0543M) and subsequently ligated at 16 °C with 50 U T4 DNA ligase (Roche, #10799009001) in a final reaction volume of 7 ml. De-crosslinking was performed with Proteinase K (0.05 µg/µl) at 65 °C and samples were then purified using phenol/chloroform extraction. The second digest was performed with 50 U Csp6I (Thermo Fisher Scientific, ER0211). Samples were ligated with 100 U T4 DNA ligase in a final volume of 14 ml and purified by precipitation in 100% ethanol. The resulting products were used directly as a PCR template for the TetO 4C viewpoint. Primers for PCR were designed according to the set-up used in Redolfi et al[2] and can be found in **Suppl. Table S3**. Library preparation was performed with the NEBNext Ultra DNA Library prep kit according to the manufacturer's instructions (NEB, E7370L). 4C-seq libraries were sequenced on an Illumina Hiseq2500 platform (50 cycles, single-end reads).

## 1.13 Cell cycle analysis of RAD21 depleted cells by flow cytometry

To validate that in auxin or dTAG-13 treated cells the distribution of cells in different stages of the cell cycle is not skewed towards cells in S and G2 phase (as cells are arrested in mitosis upon depletion of RAD21), cell cycle stage analysis was performed by flow cytometry. For this, cells were cultured until confluency on a 6-well and treated with the corresponding compound (500 µM auxin (Sigma-Aldrich, I5148-2G) or 500 nM dTAG-13 (Sigma-Aldrich, SML2601-1MG) resuspended in culturing medium) for the time indicated (0 h, 1.5 h, 2 h, 6 h). The cells were then harvested and $3 \times 10^6$ cells were fixed in 4% paraformaldehyde (EMS, 15710) at RT for 15 min. The cells were washed in PBS and stained with 5 µg/ml 4',6-Diamidino-2-phenylindole (DAPI) (D9564-10MG, Sigma-Aldrich) in 1xPBS+0.1% Triton X-100 (Sigma-Aldrich, T8787) for 30 min at RT. The cells were analyzed with a 405 nm laser line at BD LSR II SORP Analyser (BD Biosciences, BD FACSDiva™ Software v8.0.1). Distributions of cell cycle stage profiles were analyzed using FlowJo (v10, BD Biosciences).

## 1.14 Western Blot

To validate the targeted degradation of RAD21, CTCF and WAPL in the degron cell lines, cells were cultured on a 6-well to confluency and degradation was induced by adding 500 nM dTag-13 (Sigma-Aldrich, SML2601-1MG) or 500 µM auxin (Sigma-Aldrich, I5148-2G) and incubating for the indicated time (0 h, 1.5 h, 2 h, 6 h, 24 h) at 37 °C, 8% $CO_2$. The cells were washed twice with PBS and lysed in 200 µl RIPA buffer (150 mM sodium chloride, 1.0% NP-40 (Sigma-Aldrich, I8896-50ML), 0.5% sodium deoxycholate, 0.1% sodium dodecyl

sulfate (SDS), 50 mM Tris pH 8.0) incubating 1 min at 4 ℃. Lysed cells were frozen in liquid nitrogen. For the SDS-PAGE, cell lysates were thawed on ice and 2.5 U/ml SuperNuclease (Sino Biological Inc, SSNP01) was added to digest DNA for 10 min. Protein levels were quantified using the Pierce BCA protein assay (Thermo Fisher Scientific, 23225). 10 µg of protein extract were loaded onto a Mini-PROTEAN TGX Precast Gel (4-15% gradient, BioRad, 4561086) in 1xLaemmli buffer (BioRad, 1610747) containing 100 mM DTT. Samples were run at 180 V for 90 min in Tris-Glycine buffer (25 mM Tris pH 8.3, 192 mM Glycine, 0.1% (w/v) SDS). The protein was transferred to a Trans-Blot Turbo Mini 0.2 µm Nitrocellulose membrane (BioRad, 1704158) using the Trans-Blot Turbo Transfer System from BioRad. The membrane was blocked for 1 h at RT (shaking) in Odyssey Blocking Buffer (PBS) (Li-Cor Biosciences, 927-40000). The membrane was incubated with 1 µg/ml primary antibody (rabbit polyclonal anti-RAD21, abcam ab154769, rabbit polyclonal anti-WAPL, Proteintech 16370-1-AP, rabbit polyclonal anti-CTCF, Cell Signaling Technologies #2899S, rabbit anti-PK-tag (V5), abcam ab15828, and mouse monoclonal anti-Tubulin, (DM1A), Cell Signaling Technologies #3872) in Odyssey Blocking Buffer (PBS) overnight at 4 ℃. The membrane was washed three times in PBS+0.1%Tween-20 for 5 min each shaking and then incubated for 1 h at RT (shaking) with secondary antibodies (IRDye 800CW Goat anti-rabbit IgG and IRDye 680RD Goat anti-Mouse IgG, dilution 1:10,000, Li-Cor BioSciences 926-32211 and 926-68070) in Odyssey Blocking Buffer (PBS). The membrane was washed three times in PBS+0.1%Tween-20 and imaged on the Odyssey infrared imaging system (Li-Cor Biosciences).

## 1.15 Estimation of sister chromatids

To estimate the probability of encountering sister chromatids in our dual-color experiments, we manually labeled around 1400 images randomly sampled from all movies. We assigned spots to cell masks detected using CellPose. We estimated that sister chromatids occur in approx. 3% of the cells.

## 1.16 Median convergent CTCF distance

To calculate the average distance between convergent CTCF within TADs, we took the list of CTCF motifs from Nora et al.[9] The list of TADs has been taken from Zuin, Roth et al.[7] By keeping only pairs of convergent CTCF motifs within TADs, the average distance is estimated to be 140,932 bp.

## 1.17 Analysis of piggyBac insertion site mapping

To exclude reads coming from the TetR or LacI, the two ends of paired-end reads were mapped separately to the piggyBac-TetR and LacI sequences (`https://github.com/giorgettilab/Mach_et_al_chromosome_dynamics/tree/master/sequences`) using QuasR v. 1.36.0 (qAlign). Only unmapped reads were kept and mapped to the piggybac-TetO array sequence (`https://github.com/giorgettilab/Mach_et_al_chromosome_dynamics/tree/master/sequences`). Hybrid pairs with one of the read-end mapping to array were kept. The second reads from hybrid pairs were mapped to the mouse genome (build mm9) using QuasR (qAlign). Reads were then piled up in 25- bp windows using csaw v. 1.30.1 (windowCounts function). Integration sites can be identified because they correspond to local high-read coverage. Local coverage was calculated by resizing all non-zero 25 bp windows up to 525 bp (expanding by 250 bp upstream and downstream). Overlapping windows were then merged using reduce (from GenomicRanges v. 1.48.0), resulting in a set of windows wi. The size distribution of wi is multimodal, and only wi from the second mode onward were kept. For each wi we estimated the coverage ci as the number of non-zero 25 bp windows. Only wi where the coverage was >16 were considered. The exact positions of the integration sites were then identified with the center of wi.

## 1.18 Hi-C analysis

Hi-C data were analyzed using HiC-Pro version 3.1.0[10] with the –very-sensitive –end-to-end –reorder options. Only unique reads were mapped to the mouse genome (build mm9). Contact maps were combined at 8 kb with iterative correction applied afterwards. (`https://github.com/giorgettilab/Mach_et_al_chromosome_dynamics/tree/master/Hi-C`).

## 1.19 Loop analysis

Loops were called on Hi-C map of WT sample using Mustache[11] version 1.0.1 on 8 kb contact map, with p-value threshold 0.1 and sparsity threshold 0.75. Pileup analysis was performed for all samples based on WT loops using coolpup.py version 0.9.2.[12]

## 1.20 4C-Seq analysis

First, we trimmed the connector sequence, then we filtered out over-represented sequences. We mapped sequences to the mouse mm9 genome using a custom R script (`https://github.com/giorgettilab/Mach_et_al_chromosome_dynamics/`).

## 1.21 Nanopore sequencing analysis

To map nanopore sequencing reads, we first built a custom 'genome' consisting of the LacO or TetO cassette flanked by approx. 2.5 kb mouse genomic sequence upstream and downstream of the target integration site. The custom genome can be found at (`https://github.com/giorgettilab/Mach_et_al_chromosome_dynamics/tree/master/nanopore`). Analysis has been performed as in Zuin et al.[7] Briefly, reads were mapped to the custom genome using minimap2 (v. 2.17-r941) with "-x map-ont" parameter. Nanopore sequencing analysis has been implemented using Snakemake workflow (v. 3.13.3). Reads were visualized using IGV (v. 2.9.4). Full workflow can be found at `https://github.com/zhanyinx/Zuin_Roth_2021/tree/main/Nanopore`.

## 1.22 Capture-Hi-C analysis

Capture-Hi-C data were processed as in Zuin et al.[7] using HiC-Pro[10] (v. 2.11.4). Briefly, read pairs were mapped to the mouse genome (build mm9). Chimeric reads were recovered after recognition of the ligation site. Only unique valid pairs mapping to the target regions were used to build contact maps. Iterative correction (ICE)[13] was then applied on binned data. The target regions can be found at `https://github.com/zhanyinx/Zuin_Roth_2021`.

## 1.23 Differential Capture-Hi-C maps

We accounted for differences in genomic distances due to the presence of the ectopic sequence when evaluating the structural perturbation induced by the insertion of the TetO and LacO arrays. To account for these differences, we generated distance-normalized capture-Hi-C maps where each entry corresponds to the interaction normalized by the corrected genomic distance between the interacting bins. We then calculated the WT-allele corrected ratios between distance normalized maps using the following formula:

$$ratio = \frac{2 \cdot mut - wt}{wt} \tag{1}$$

Where $mut$ is the distance normalized heatmap of the line with TetO or LacO arrays and $wt$ is the distance normalized heatmap of the wild-type. A bilinear smoothing with a window of 2 bins has been applied to the

ratio maps to evaluate the structural perturbation induced by the insertion of the arrays. To quantify the effect of ectopic CTCF sites, we followed the same approach as the quantification of the arrays insertion with the exception that the ratio is calculated using the following formula

$$ratio = \frac{2 \cdot mut_{+} - WT}{2 \cdot mut_{-} - WT} \tag{2}$$

Where $mut_{+}$ and $mut_{-}$ represent the lines with and without the ectopic CTCF (or promoter), respectively.

## 1.24 Conversion of simulation steps to real time

In order to convert the timescales between experiments and simulations, we measured the time needed for a bead (and 8 kb TetO array) to move for its own size (95nm = 15nm x $\sqrt{40}$ = estimated size of a nucleosome x estimated number of nucleosomes in each 8 kb segment, assuming that chromatin inside each 8 kb behave as an ideal chain; this is in line with the previous estimations for a 3 kb segment[14]). To this aim, 2D movies acquired in cells with randomly inserted TetOs every 0.1 s for 10 s were analyzed with the same strategy as in **Multi-operator image analysis and localisation error estimation**, now without 3D linkage. We used the MSD curve to estimate the time necessary to move for 95 nm for each line. We used the average value across 3 lines to convert the timescales between experiments and simulations.

In simulations, a bead needs approx. 1800 simulation steps to move of its own size, whereas in experiment this time is estimated to be approx. 0.86 s. Therefore 1 s of the real time corresponds to approx. 2100 simulation steps. This approximation neglects the fact that the coarse-grained bead describes a segment of the fiber that in the cell can display internal dynamics, and the length represented by the bead can be variable. We assumed that such internal dynamics is faster and uncorrelated to the motion we want to simulate. Thus each bead is characterized only by its average size, that is 95 nm, neglecting the fluctuations around the average.

## 1.25 Metrics for quantifying similarity between simulations and experiments

To evaluate the similarity between experimental features (f) (here: contact duration, probability of being in the proximal state, fraction of time spent in the proximal state) we used the relative difference ($RD$) defined as:

$$RD = \frac{\mid f_{exp} - f_{sim} \mid}{f_{exp}} \tag{3}$$

To find the set of loop extrusion parameters that best reproduce the experiments in the presence of cohesin, we used the sum of the relative difference of the following features: gaussian mean of contact state, the contact duration and the fraction of time spent in the looped state in the $\pm$ CTCF. To find the set of loop extrusion parameters that best reproduce the experiments in the absence of cohesin, we decreased the loading rate to reach extruder densities of 0.87 per Mb while keeping the extruder residence time constant (5.5 min).

# 2 Polymer modelling

## 2.1 Introduction

The theory of anomalous diffusion was developed studying the dynamics of loci on a polymer chain with respect to a fixed reference system. In our experimental work we measure distances between pairs of genomic locations (Figs. 3, 4, 5). We show in the following that one can extract the scaling exponent that characterises subdiffusion from the dynamics of the distance between pairs of loci.

We first discuss the subdiffusive motion of the difference vector between two beads of a Rouse chain, that can be solved analytically and gives for short times the standard scaling exponent $1/2$ as that of the position of a bead. Then we discuss the dynamics of the scalar distance between two beads, showing numerically that for short times it gives the same scaling exponent as the vector distance.

Finally, we show that in presence of excluded volume the scaling exponent is larger than the one predicted for the Rouse chain.

## 2.2 Subdiffusion of the difference vector in the Rouse chain

Consider a standard Gaussian chain of $N$ links. In the continuous limit, the position $\mathbf{r}(x,t)$ of a locus on the chain evolves according to the diffusion equation

$$\xi \frac{\partial \mathbf{r}(x,t)}{\partial t} = \mathbf{f}^r(x,t) + \frac{3T}{a^2}\frac{\partial^2 \mathbf{r}(x,t)}{\partial x^2}, \tag{4}$$

where $x \in [0, N]$ labels the position along the chain and is assumed to be continuous, $a$ is the distance between neighbouring beads and is thus assumed small, $T$ is the temperature, $\xi$ is the friction coefficient and $\mathbf{f}^r$ is the random force with zero mean and correlations

$$\langle f^r_\alpha(x,t) \cdot f^r_\beta(x',t')\rangle = 2\xi T \delta(x-x')\delta(t-t')\delta_{\alpha\beta}. \tag{5}$$

Following the standard solution of the Rouse chain (see, e.g., A.Yu. Grosberg and A.R. Khokhlov, *Statistical Physics of Macromolecules*, AIP-Press, 1994), let's write the position of a bead on the chain as a Fourier series

$$\mathbf{r}(x,t) = y_0(t) + 2\sum_{p=1}^{\infty} y_p(t)\cos\left(\frac{\pi p x}{N}\right). \tag{6}$$

where $p = 0, 1, 2...$ The $y_p$ coordinates are called *Rouse Modes* and are obtained by the inverse Fourier transform

$$y_p(t) = \frac{1}{N}\int_0^N dx \cos\left(\frac{\pi p x}{N}\right)\mathbf{r}(x,t). \tag{7}$$

Let's briefly discuss the mean square displacement of a single bead. This can be written as

$$\langle(\mathbf{r}(x,t) - \mathbf{r}(x,0))^2\rangle = \frac{6T}{N\xi}t + \frac{4Na^2}{\pi^2}\sum_{p=1}^{\infty}\frac{1}{p^2}\cos^2\left(\frac{\pi p x}{N}\right)\left(1 - e^{-t/\tau_p}\right). \tag{8}$$

where $\tau_p \equiv \frac{N^2 a^2 \xi}{3\pi^2 T p^2}$ is the Rouse relaxation time for mode $p$; in particular, $\tau_1$ is the slowest relaxation mode.

For large times $t \gg \tau_1$ the second term in (8) is negligible and the mean square displacement of a bead is the same as that of the whole coil. On the other hand, when $t \ll \tau_1$ the large values of $p$ are dominant and the sum can be approximated by an integral. Moreover, if we consider beads far from the ends of the chain, the

function $\cos^2(\frac{\pi p x}{N})$ is fast oscillating and can be substituted by its mean value $\frac{1}{2}$. In this limit, the mean square displacement of a bead is approximately

$$\langle(\mathbf{r}(x,t)-\mathbf{r}(x,0))^2\rangle \approx \frac{2\sqrt{3}a}{\sqrt{\pi}}\sqrt{\frac{T}{\xi}}\sqrt{t}. \tag{9}$$

Now, we want to compute the mean square displacement of the difference vector between two beads

$$\Delta(t) = \big\langle(\boldsymbol{\delta}(t)-\boldsymbol{\delta}(0))^2\big\rangle, \tag{10}$$

where $\boldsymbol{\delta}(t) = \mathbf{r}(x,t)-\mathbf{r}(y,t)$. Then, using Eq. (6) we have

$$\begin{aligned}\Delta(t) &= \langle \mathbf{r}(x,t)-\mathbf{r}(y,t)-\mathbf{r}(x,0)+\mathbf{r}(y,0)\rangle \\ &= 4\sum_{p=1}^{\infty}\Big(\cos(\frac{\pi p x}{N})-\cos(\frac{\pi p y}{N})\Big)^2\langle(y_p(t)-y_p(0))^2\rangle\end{aligned} \tag{11}$$

But

$$\begin{aligned}\langle(y_p(t)-y_p(0))^2\rangle &= \langle y_p^2(t)\rangle+\langle y_p^2(0)\rangle-2\langle y_p(t)\cdot y_p(0)\rangle \\ &= \frac{3T\tau_p}{N\xi}(1-e^{-t/\tau_p})\end{aligned} \tag{12}$$

where we used $\langle y_p(t)\cdot y_p(t')\rangle = \frac{3T\tau_p}{2N\xi}e^{-(t-t')/\tau_p}$.

Combining Eqs. (11) and (12) we get

$$\begin{aligned}\Delta(t) &= \frac{4Na^2}{\pi^2}\sum_{p=1}^{\infty}\frac{1}{p^2}\Big(\cos\frac{\pi p x}{N}-\cos\frac{\pi p y}{N}\Big)^2\Big(1-e^{-t/\tau_p}\Big) \\ &= \frac{4Na^2}{\pi^2}\sum_{p=1}^{\infty}\frac{4}{p^2}\sin^2\Big(\frac{\pi p}{2N}(L+2x)\Big)\sin^2\Big(\frac{\pi p}{2N}L\Big)\Big(1-e^{-t/\tau_p}\Big),\end{aligned} \tag{13}$$

where in the last step we used prosthaphaeresis formulae and defined $L = |x-y|$ as the genomic distance. In the continuous limit, with the same approximations employed to obtain eq. (9), we substitute the sum with an integral and the former $sin$ function with its mean value $\frac{1}{2}$. Eventually,

$$\begin{aligned}\Delta(t) &= \frac{8Na^2}{\pi^2}\int_0^{\infty}\frac{1}{p^2}\sin^2\Big(\frac{\pi p}{2N}L\Big)\Big(1-e^{-\frac{3\pi^2 T t}{N^2a^2\xi}p^2}\Big) \\ &= \frac{4\sqrt{3}a}{\sqrt{\pi}}\sqrt{\frac{T}{\xi}}\sqrt{t}\Big(1-e^{-\tau/t}\Big)+2La^2\,\text{erfc}\Big(\frac{La\sqrt{\xi}}{\sqrt{12}\sqrt{T}}\frac{1}{\sqrt{t}}\Big),\end{aligned} \tag{14}$$

where we defined the crossover time $\tau = \frac{L^2a^2\xi}{12T}$. In the limit of large times $t \gg \tau$ the first term is negligible and the mean square displacement of the distance between two beads approaches

$$\Delta(t) \approx 2La^2, \tag{15}$$

corresponding to a plateau. In fact, this value is independent on time for times beyond $\tau$ and depends on the genomic distance $L$ between the two sites, taking a value that is twice the steady-state mean squared distance between two beads on the chain. Noticeably, also the crossover time $\tau$ depends on the genomic distance $L$, increasing quadratically with it. For long times the correlations between the two beads grow to compensate the sub–diffusion term; the correlations arise from the fact that on this time scale the two beads diffuse as a whole

together with the beads laying between them. On the other hand, when $t \ll \tau$,

$$\Delta(t) \approx \frac{4\sqrt{3}a}{\sqrt{\pi}}\sqrt{\frac{T}{\xi}} \cdot \sqrt{t} \tag{16}$$

that is the same as the sub–diffusion of the single bead and reflect the initial regime when the two beads move independently on each other. The coefficient $\frac{4\sqrt{3}a}{\sqrt{\pi}}\sqrt{\frac{T}{\xi}}$ is exactly double the coefficient of the MSD of a single bead for short times (see eq. (9)), as expected.

## 2.3 Subdiffusion of the distance

The quantity that is directly accessible in dual colour experiments is not the difference vector but the distance $d(t) = |\boldsymbol{\delta}(t)|$ and in particular

$$\Delta'(t) = \langle (d(t) - d(0))^2 \rangle = \langle |\boldsymbol{\delta}(t)|^2 \rangle + \langle |\boldsymbol{\delta}(0)|^2 \rangle - 2\langle |\boldsymbol{\delta}(t)| \cdot |\boldsymbol{\delta}(0)| \rangle \tag{17}$$

which is different from

$$\Delta(t) = \left\langle (\boldsymbol{\delta}(t) - \boldsymbol{\delta}(0))^2 \right\rangle = \langle |\boldsymbol{\delta}(t)|^2 \rangle + \langle |\boldsymbol{\delta}(0)|^2 \rangle - 2\langle \boldsymbol{\delta}(t) \cdot \boldsymbol{\delta}(0) \rangle \tag{18}$$

because it contains the correlation between two scalars instead of a scalar product.

The difference between Eqs. (17) and (18) is the last term, that is the correlation function of either the displacement vector or the displaced distance to the initial ones. From the definition of scalar product, $\langle \boldsymbol{\delta}(t) \cdot \boldsymbol{\delta}(0) \rangle = \langle |\boldsymbol{\delta}(t)| \cdot |\boldsymbol{\delta}(0)| \cdot \cos\theta(t) \rangle$, where $\theta(t)$ is the angle between $\boldsymbol{\delta}(t)$ and $\boldsymbol{\delta}(0)$. The rotational diffusion of the direction defined by $\boldsymbol{\delta}(t)$ takes place with a diffusion coefficient $D_{rot} = 2T/\xi d^2$ (see Cugliandolo et al.[15]), that is on an elementary timescale of the order of $\xi a^2 L/2T$. On the other hand, the motion of the distance $d(t)$ takes place, for $t < \tau$, on an elementary timescale $\xi a^2/2T$. Thus, one expects that for $L \gg 1$, $\cos\theta(t)$ is weakly dependent on time and the two correlation functions are different for a nearly constant quantity. In conclusion, $\Delta(t)$ and $\Delta'(t)$ are expected to show the same scaling behaviour at short times.

To substantiate these expectations, we have calculated $\Delta(t)$ and $\Delta'(t)$ for two beads ($L = |x - y| = 100$) of a Rouse chain (of length $N = 1000$). The results are displayed in Fig. 1. Both curves display the expected exponent $\approx 1/2$ in the initial linear part.

Also in the case of a bead-spring chain with excluded volume, the initial scaling of the difference vector is the same as that of the distance (see Fig. 2 and text below).

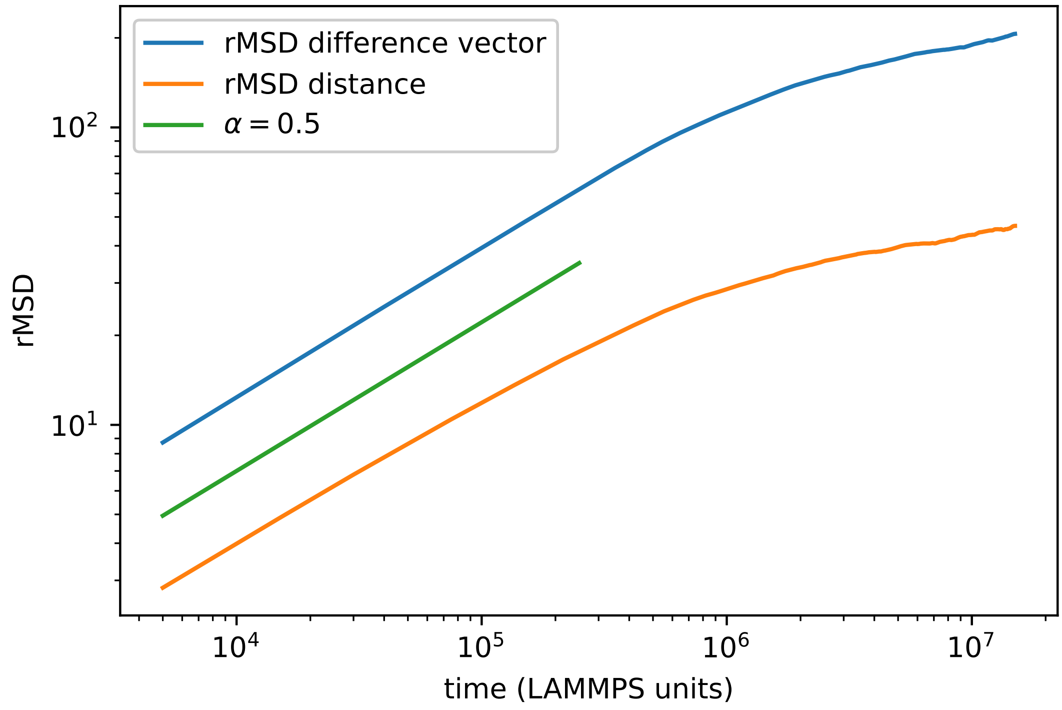


Figure 1: The subdiffusion $\Delta(t)$ of the difference vector between two beads separated by 100 other beads in a Rouse chain and of that $\Delta'(t)$ of the distance between them.

### 2.4 Bead-spring chain with excluded volume

A scaling argument can be used to show that adding excluded volume to a Rouse chain, the scaling exponent of the sub-diffusive regime increases to $6/11 \approx 0.55$ (see Tamm et al.[16]).

In fact, one can imagine to split the chain at time $t$ into domains of size $g(t)$, like blobs that moves independently on each other. The genomic distance associated to $g(t)$ is $L(t)$, that is the number of monomers moving coherently in the domain; to be able to define a domain as something that moves independently on the other domains on the time scale $t$, its spatial size $g(t)$ must be of order of the displacement of a monomer over a time $t$,

$$g(t) \sim x(t). \tag{19}$$

At the same time, the displacement of a single monomer has the same behaviour as that of the domain it belongs to; such a domain diffuses like a Brownian particle but with a diffusion coefficient which is inversely proportional to the number of monomers $L(t)$ (assuming that the thermal forces acting on the monomers are independent on each other), thus one has

$$x^2(t) \sim \frac{t}{L(t)}. \tag{20}$$

For a chain with excluded volumes the size of a domain of $L$ monomers has typical size

$$g \sim aL^{3/5} \tag{21}$$

and, substituting Eqs. (20) and (21) into Eq. (19), one obtains

$$L^{3/5}(t) \sim a\frac{t^{1/2}}{L^{1/2}(t)} \;\rightarrow\; L(t) \sim t^{5/11}. \tag{22}$$

Finally, Eq. (20) can be rewritten as

$$x^2(t) \sim t^{6/11} \approx t^{0.55}. \tag{23}$$

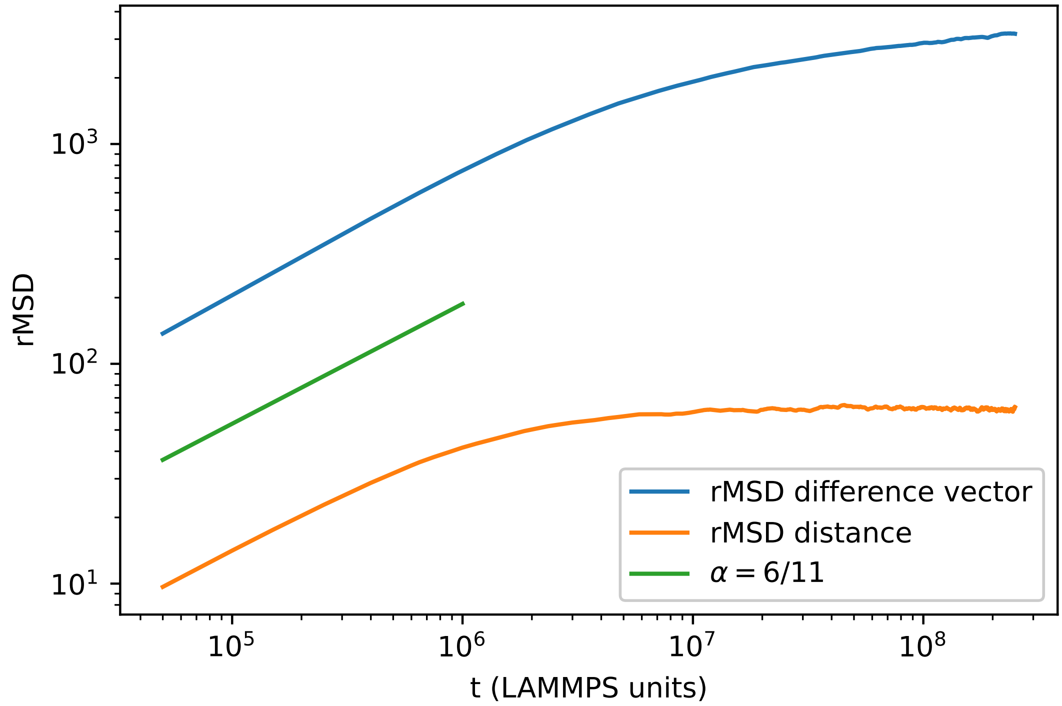


Figure 2: The subdiffusion $\Delta(t)$ of the difference vector between two beads separated by 100 other beads in a bead-spring chain with excluded volume and of that $\Delta'(t)$ of the distance between them. The excluded volumes are implemented by the repulsive–only Lennard–Jones potential.

Following the arguments of the previous sections, we should thus expect a power law scaling $rMSD \sim t^{6/11}$ of the pairwise distance between two beads in the case of a chain with excluded volumes, in the small times regime where the saturation is not yet occurring. This behaviour is quite evident in Fig. 2 for both the distance and difference vector curves.

# 3 Supplementary Figures

## 3.1 Supplementary Figure 1: Features of HMM states called on simulations across the parameter space

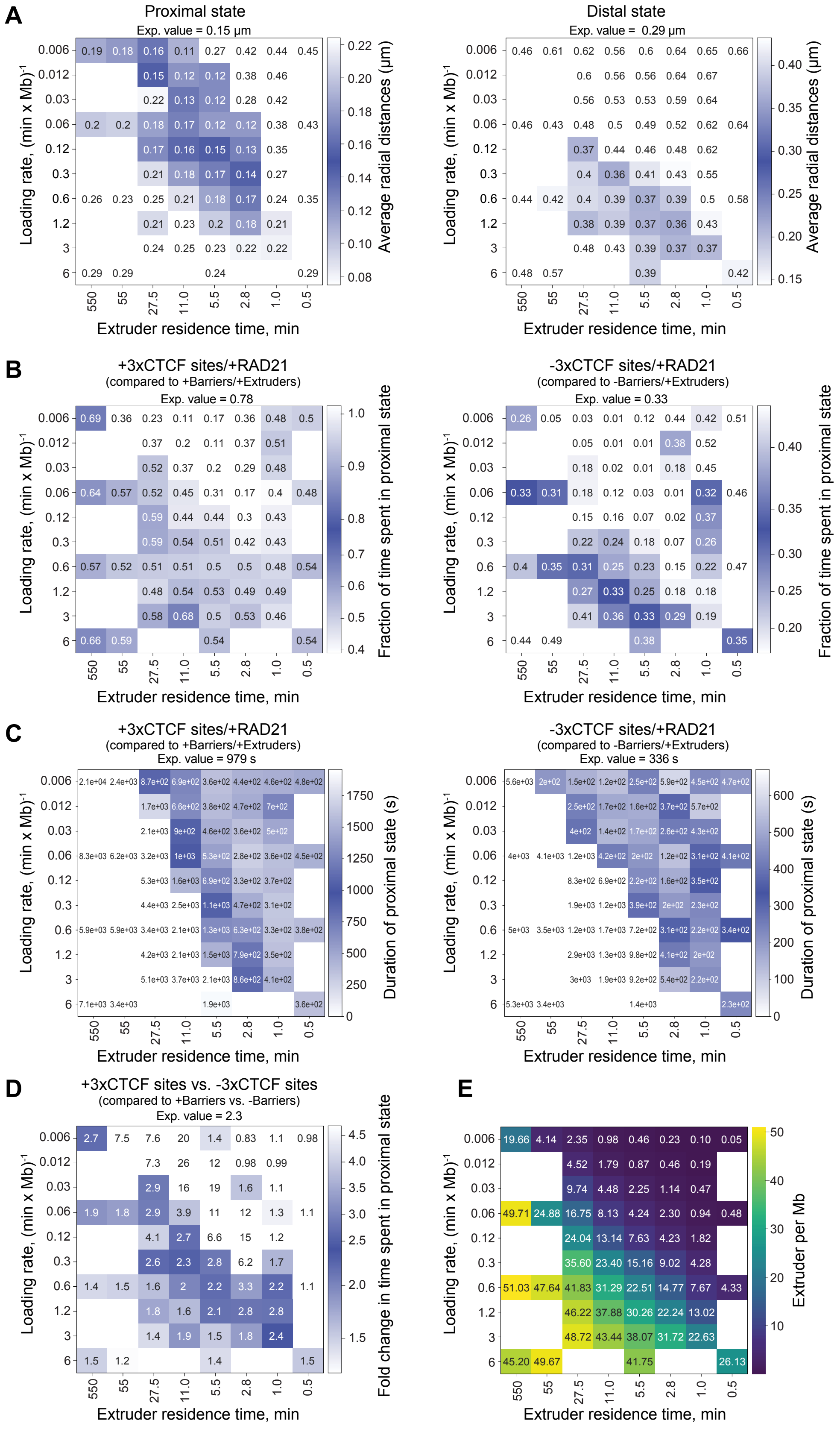

A. Heatmap showing the agreement of average radial distances of either the proximal state (left) or the distal state (right) called by HMM on all simulated systems of extrusion speed 1 kb/s with the proximal state or

B. distal state called by HMM on the experimental data. Darker shades of blue indicate better agreement with experimental values.

C. Same as in panel A, but for the fraction of time spent in the proximal state (left) and distal state (right).

D. Same as in panel A but for the duration of the proximal state for either the condition +Barriers/+3xCTCF sites (left) or -Barriers/-3xCTCF sites (right) in the presence of the Extruders/RAD21.

E. Same as in panel A but for the fold change of time spent in a proximal state called by HMM on the experimental data for the comparison of the condition +Barriers/+3xCTCF sites vs. -Barriers/-3xCTCF sites in the presence of Extruders/RAD21.

F. Extruder densities per Mb for all simulated systems with extrusion speed 1 kb/s. Color-coded for the density per Mb.

# 4 Supplementary Videos

## 4.1 Supplementary Video S1: Live-cell imaging of TetO arrays upon depletion of RAD21

Time course of RAD21 degradation upon induction with 500 µM auxin in RAD21-AID-eGFP cells. TetO integrations are tagged with TetR-tdTomato (magenta) and RAD21 is tagged with eGFP (green). Green fluorescence is lost within 90 min after induction of degradation (exposure time (eGFP) = 50 ms, exposure time (tdTomato) = 50 ms, deconvolved, max. intensity projection, duration of movie 30 min, dt=10 s). *File is provided as a separate MPG file.*

## 4.2 Supplementary Video S2: Dynamics of LacO-TetO radial distances

Representative movie of dual-color imaging of LacO (green) and TetO (magenta) arrays flanked by 3xCTCF sites integrated on chromosome 15 at a distance of 150 kb (exposure time (eGFP) = 50 ms, exposure time (tdTomato) = 50 ms, deconvolved, max. intensity projection, duration of movie 1 h, dt=30 s). *File is provided as a separate MPG file.*

## 4.3 Supplementary Video S3: Cohesin and CTCF decrease average LacO-TetO radial distances

Representative movies of dual-color imaging of LacO (green) and TetO (magenta) arrays on chromosome 15 at a distance of 150 kb. Left panel: Cell line with 3xCTCF sites flanking LacO and TetO (in the presence of RAD21); middle panel: Cell line where 3xCTCF sites have been removed (in the presence of RAD21); Right panel: Cell line with 3xCTCF sites flanking the array, but where RAD21 has been degraded with 500 nM dTAG-13 (exposure time (eGFP) = 50 ms, exposure time (tdTomato) = 50 ms, deconvolved, max. intensity projection, duration of movie 1 h, dt=30 s). *File is provided as a separate MPG file.*

# 5 Supplementary Tables

## 5.1 Supplementary Table S1: Statistics of live-cell imaging data

Number of independent clonal lines, the number of cells imaged, and the number of tracks extracted from the movies for each condition tested in the live-cell imaging experiments. The number of tracks represent independent measurements for each condition. *File is provided as a separate XLS file.*

## 5.2 Supplementary Table S2: Statistical tests for duration and frequency of the HMM-called states

P-values based on two-sided Student t-tests for the comparison of duration and frequency of the proximal state between the different conditions from experimental live-cell imaging data and simulated data; based on the HMM-calls. *File is provided as a separate XLS file.*

## 5.3 Supplementary Table S3: Oligonucleotides

List of oligonucleotide sequences used in this study, including primer sequences used for cloning and genotyping of cell lines, gRNA sequences for Nanopore sequencing, capture probes for PiggyBac insertion site mapping and 4C-seq primers. *File is provided as a separate XLS file.*

## 5.4 Supplementary Table S4: Spot detection and tracking parameters

List of spot detection and tracking parameters used for each live-cell imaging dataset, including names of the deepBlink model applied, spot and tracking parameters used in Fiji and motion-correction parameters applied. *File is provided as a separate XLS file.*

# 6 Flow cytometry gating strategy

## A - Cell cycle stage analysis of Rad21-AID-eGFP cells after induction of Rad21 depletion with 500 µM auxin

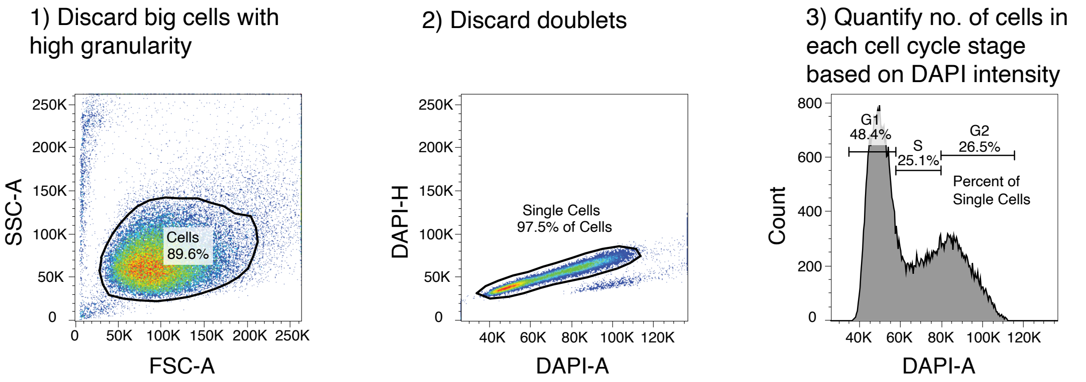


| Sample | Rad21-AID-eGFP untreated | Rad21-AID-eGFP 90min | Rad21-AID-eGFP 360min |
|---|---|---|---|
| Count of Events (Cells) | 52032 | 50946 | 51517 |
| Count of Events (Single cells) | 50710 | 49899 | 49634 |
| Percent of Parent (Single Cells) | 97.5 | 97.9 | 96.3 |
| Count of Events (G1) | 24562 | 21683 | 7688 |
| Percent of Parent (G1) | 48.4 | 43.5 | 15.5 |
| Count of Events (S) | 12709 | 12931 | 12112 |
| Percent of Parent (S) | 25.1 | 25.9 | 24.4 |
| Count of Events (G2) | 13439 | 15285 | 29834 |
| Percent of Parent (G2) | 26.5 | 30.6 | 60.1 |

Gates for all three conditions were the same.

## B - Cell cycle stage analysis of Rad21-HaloTag-FKBP cells after induction of Rad21 depletion with 500 nM dTag-13

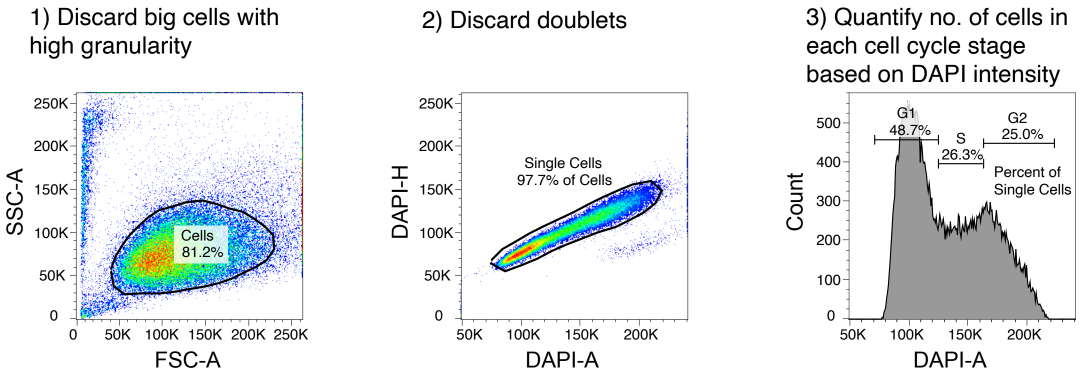


| Sample | Rad21-HaloTag-FKBP untreated | Rad21-HaloTag-FKBP 2h | Rad21-HaloTag-FKBP 6h |
|---|---|---|---|
| Count of Events (Cells) | 49836 | 50093 | 50311 |
| Count of Events (Single cells) | 48685 | 48511 | 47344 |
| Percent of Parent (Single Cells) | 97.7 | 96.8 | 94.1 |
| Count of Events (G1) | 23708 | 21057 | 10845 |
| Percent of Parent (G1) | 48.7 | 43.4 | 22.9 |
| Count of Events (S) | 12803 | 12861 | 12478 |
| Percent of Parent (S) | 26.3 | 26.5 | 26.4 |
| Count of Events (G2) | 12174 | 14593 | 24021 |
| Percent of Parent (G2) | 25 | 30.1 | 50.7 |

Gates for all three conditions were the same.

SSC-A: Side scatter amplitude
FSC-A: Forward scatter amplitude
DAPI-H: DAPI height
DAPI-A: DAPI amplitude

## References


[1] O. Masui, I. Bonnet, P. Le Baccon, I. Brito, T. Pollex, N. Murphy, P. Hupé, E. Barillot, A. S. Belmont, and E. Heard. Live-cell chromosome dynamics and outcome of X chromosome pairing events during ES cell differentiation. *Cell*, 145(3):447–58, 2011.

[2] J. Redolfi, Y. Zhan, C. Valdes-Quezada, M. Kryzhanovska, I. Guerreiro, V. Iesmantavicius, T. Pollex, R. S. Grand, E. Mulugeta, J. Kind, G. Tiana, S. A. Smallwood, W. de Laat, and L. Giorgetti. DamC reveals principles of chromatin folding in vivo without crosslinking and ligation. *Nat Struct Mol Biol*, 26(6):471–480, 2019.

[3] T. Pollex and E. Heard. Nuclear positioning and pairing of X-chromosome inactivation centers are not primary determinants during initiation of random X-inactivation. *Nat Genet*, 51(2):285–295, 2019.

[4] I. F. Lau, S. R. Filipe, B. Søballe, O. A. Økstad, F. X. Barre, and D. J. Sherratt. Spatial and temporal organization of replicating escherichia coli chromosomes. *Mol Microbiol*, 49(3):731–43, 2003.

[5] N. Q. Liu, M. Maresca, T. van den Brand, L. Braccioli, Mmga Schijns, H. Teunissen, B. G. Bruneau, E. P. Nora, and E. de Wit. WAPL maintains a cohesin loading cycle to preserve cell-type-specific distal gene regulation. *Nat Genet*, 53(1):100–109, 2021.

[6] J. B. Grimm, A. K. Muthusamy, Y. Liang, T. A. Brown, W. C. Lemon, R. Patel, R. Lu, J. J. Macklin, P. J. Keller, N. Ji, and L. D. Lavis. A general method to fine-tune fluorophores for live-cell and in vivo imaging. *Nat Methods*, 14(10):987–994, 2017.

[7] J. Zuin, G. Roth, Y. Zhan, J. Cramard, J. Redolfi, E. Piskadlo, P. Mach, M. Kryzhanovska, G. Tihanyi, H. Kohler, M. Eder, C. Leemans, B. van Steensel, P. Meister, S. Smallwood, and L. Giorgetti. Nonlinear control of transcription through enhancer-promoter interactions. *Nature*, 604(7906):571–577, 2022.

[8] E. Splinter, E. de Wit, H. J. van de Werken, P. Klous, and W. de Laat. Determining long-range chromatin interactions for selected genomic sites using 4C-seq technology: from fixation to computation. *Methods*, 58(3):221–30, 2012.

[9] E. P. Nora, A. Goloborodko, A. L. Valton, J. H. Gibcus, A. Uebersohn, N. Abdennur, J. Dekker, L. A. Mirny, and B. G. Bruneau. Targeted degradation of CTCF decouples local insulation of chromosome domains from genomic compartmentalization. *Cell*, 169(5):930–944.e22, 2017.

[10] N. Servant, N. Varoquaux, B. R. Lajoie, E. Viara, C. J. Chen, J. P. Vert, E. Heard, J. Dekker, and E. Barillot. Hic-pro: an optimized and flexible pipeline for hi-c data processing. *Genome Biol*, 16:259, 2015.

[11] A. Roayaei Ardakany, H. T. Gezer, S. Lonardi, and F. Ay. Mustache: multi-scale detection of chromatin loops from Hi-C and Micro-C maps using scale-space representation. *Genome Biol*, 21(1):256, 2020.

[12] I. M. Flyamer, R. S. Illingworth, and W. A. Bickmore. Coolpup.py: versatile pile-up analysis of Hi-C data. *Bioinformatics*, 36(10):2980–2985, 2020.

[13] M. Imakaev, G. Fudenberg, R. P. McCord, N. Naumova, A. Goloborodko, B. R. Lajoie, J. Dekker, and L. A. Mirny. Iterative correction of Hi-C data reveals hallmarks of chromosome organization. *Nat Methods*, 9(10):999–1003, 2012.

[14] L. Giorgetti, R. Galupa, E. P. Nora, T. Piolot, F. Lam, J. Dekker, G. Tiana, and E. Heard. Predictive polymer modeling reveals coupled fluctuations in chromosome conformation and transcription. *Cell*, 157(4):950–63, 2014.

[15] L. F. Cugliandolo, G. Gonnella, and A. Suma. Rotational and translational diffusion in an interacting active dumbbell system. *Phys Rev E Stat Nonlin Soft Matter Phys*, 91(6):062124, 2015.

[16] M. V. Tamm and K. Polovnikov. Dynamics of Polymers: Classic Results and Recent Developments. *Order, disorder and criticality: Advanced problems of phase transition theory*, pages 113–172, 2018.
