# Supplementary material for: Cohesin and CTCF control the dynamics of chromosome folding
Source: Nat Genet. 2022 Dec 5;54(12):1907–18. doi: 10.1038/s41588-022-01232-7 (PMC9729113; doi:10.1038/s41588-022-01232-7)
Supplement: Supplementary file 2 — Reporting Summary [file 41588_2022_1232_MOESM2_ESM.pdf]

nature portfolio

Corresponding author(s): Luca Giorgetti

Last updated by author(s): Oct 3, 2022

Reporting Summary

Nature Portfolio wishes to improve the reproducibility of the work that we publish. This form provides structure for consistency and transparency in reporting. For further information on Nature Portfolio policies, see our Editorial Policies and the Editorial Policy Checklist.

Statistics

For all statistical analyses, confirm that the following items are present in the figure legend, table legend, main text, or Methods section.

n/a	Confirmed	
☐	☒	The exact sample size (*n*) for each experimental group/condition, given as a discrete number and unit of measurement
☐	☒	A statement on whether measurements were taken from distinct samples or whether the same sample was measured repeatedly
☐	☒	The statistical test(s) used AND whether they are one- or two-sided<br>*Only common tests should be described solely by name; describe more complex techniques in the Methods section.*
☐	☒	A description of all covariates tested
☐	☒	A description of any assumptions or corrections, such as tests of normality and adjustment for multiple comparisons
☐	☒	A full description of the statistical parameters including central tendency (e.g. means) or other basic estimates (e.g. regression coefficient) AND variation (e.g. standard deviation) or associated estimates of uncertainty (e.g. confidence intervals)
☐	☒	For null hypothesis testing, the test statistic (e.g. *F*, *t*, *r*) with confidence intervals, effect sizes, degrees of freedom and *P* value noted<br>*Give P values as exact values whenever suitable.*
☒	☐	For Bayesian analysis, information on the choice of priors and Markov chain Monte Carlo settings
☒	☐	For hierarchical and complex designs, identification of the appropriate level for tests and full reporting of outcomes
☒	☐	Estimates of effect sizes (e.g. Cohen's *d*, Pearson's *r*), indicating how they were calculated

Our web collection on statistics for biologists contains articles on many of the points above.

Software and code

Policy information about availability of computer code

Data collection	BD LSRII SORP Analyser was used for acquiring DAPI intensity by flow cytometry, BD Influx cell sorter was used for the FACS, MinION (protocol: SQL-CAS109) was used for Nanopore sequencing, Illumina Nextseq500 platform was used for Hi-C, Capture-C and integration site mapping, Illumina Hiseq2500 platform was used for 4C-seq. Odyssey infrared imaging system (Li-Cor Biosciences) was used for imaging Western Blot membranes. Live-cell imaging was performed on a Nikon Eclipse Ti-E inverted wide-field microscope with a Total Internal Reflection Microscopy iLAS2 module. Agarose gels were visualized using a Typhoon FLA 9500 scanner (GE Healthcare).
Data analysis	minimap2 (v. 2.17-r941), Snakemake (v. 3.13.3), IGV (v. 2.9.4), HiC-Pro (v. 2.11.4 for capture analysis v. 3.1.0 for Hi-C), Fiji (v. 2.0), TrackMate (v. 6.0.0), Trackpy (v 0.5.0), CellPose (v 0.6.5), deepBlink (0.1.1), scipy (v.1.4.1), Mustache(v. 1.0.1), coolpup.py (v. 0.9.2), PyMOL (v. 2.3.3), FlowJo (v10, BD Biosciences), BD FACSDiva (v8.0.1., BD Biosciences), Visiview (4.4.0.12, Visitron), Huygens Remote Manager (v3.8), QuasR (v1.36.0), csaw (v 1.30.1), GenomicRanges (v 1.48.0)<br>Custom codes can be found in<br>https://github.com/zhanyinx/SPT_analysis/<br>https://github.com/polly-code/lammps_le/<br>https://github.com/giorgettilab/Mach_et_al_chromosome_dynamics/<br>https://github.com/zhanyinx/hmmlearn/

For manuscripts utilizing custom algorithms or software that are central to the research but not yet described in published literature, software must be made available to editors and reviewers. We strongly encourage code deposition in a community repository (e.g. GitHub). See the Nature Portfolio guidelines for submitting code & software for further information.

Data

Policy information about availability of data

All manuscripts must include a data availability statement. This statement should provide the following information, where applicable:

- Accession codes, unique identifiers, or web links for publicly available datasets
- A description of any restrictions on data availability
- For clinical datasets or third party data, please ensure that the statement adheres to our policy

The image tracking data was uploaded to Zenodo (https://doi.org/10.5281/zenodo.7127868). All capture-C, Hi-C, 4C, integration site mapping sequencing fastq files generated in this study have been uploaded to the Gene Expression Omnibus (GEO) under accession GSE197238 (https://www.ncbi.nlm.nih.gov/geo/query/acc.cgi?acc=GSE197238). The following public databases were used: BSgenome.Mmusculus.UCSC.mm9 (https://bioconductor.org/packages/release/data/annotation/html/BSgenome.Mmusculus.UCSC.mm9.html), Supplemental Information to Nora, Elphège P., et al. "Targeted degradation of CTCF decouples local insulation of chromosome domains from genomic compartmentalization." Cell 169.5 (2017): 930-944. Data for all plots for the Figures and Extended Data shown as well as original image files for gels and blots can be found in the Source Data section.

Field-specific reporting

Please select the one below that is the best fit for your research. If you are not sure, read the appropriate sections before making your selection.

☒ Life sciences ☐ Behavioural & social sciences ☐ Ecological, evolutionary & environmental sciences

For a reference copy of the document with all sections, see nature.com/documents/nr-reporting-summary-flat.pdf

Life sciences study design

All studies must disclose on these points even when the disclosure is negative.

Sample size	No statistical methods were applied to predetermine sample size for live-cell imaging experiments. For live-cell imaging experiments of random TetO integrations, 3-4 biological replicates were performed with 1-2 technical replicates each. This resulted on average in 267 cells with 8402 trajectories analyzed per condition. For live-cell imaging of the dual-array cell lines, 3-7 biological replicates including 4 technical replicates each were performed resulting in on average 220 cells/condition analyzed. For capture-C, Hi-C, 4C-seq, piggybac insertion site mapping and Nanopore sequencing with Cas9-guided adapter ligation 1 biological replicate was performed following the standard in the field on no. of reads sequenced. For flow cytometry measurements 2 biology replicates were performed recording >50,000 events for each condition. Western Blot analysis and genotyping PCR with subsequent agarose gel electrophoresis was performed with 1-2 biological and 2 technical replicates. Number of replicates was chosen based on standards in the field.
Data exclusions	No data was excluded from the analysis.
Replication	Live-cell imaging experiments were performed in 3-7 biological replicates and all replicates showed consistent results. For capture-C, Hi-C, 4C-seq, piggybac insertion site mapping and Nanopore sequencing with Cas9-guided adapter ligation 1 biological replicate was performed. For flow cytometry measurements 2 biology replicates were performed. Western Blot analysis and genotyping PCR with subsequent agarose gel electrophoresis was performed with 1-2 biological and 2 technical replicates.
Randomization	No randomization was performed as the study did not require sample allocation into different groups. Experimental groups were defined by the genotype of the cell line used and samples, i.e. cells measured, were chosen at random.
Blinding	Blinding was not possible for data collection in live-cell imaging experiments, as data acquisition required identification of the sample for further processing. Data analysis for live-cell imaging, capture-C, Hi-C, 4C-seq and Piggybac insertion site mapping were performed in a blinded manner. Blinding was not necessary for the other experiments since the results are quantitative and did not require subjective judgment or interpretation.

Reporting for specific materials, systems and methods

We require information from authors about some types of materials, experimental systems and methods used in many studies. Here, indicate whether each material, system or method listed is relevant to your study. If you are not sure if a list item applies to your research, read the appropriate section before selecting a response.

Materials & experimental systems

n/a	Involved in the study
☐	☒ Antibodies
☐	☒ Eukaryotic cell lines
☒	☐ Palaeontology and archaeology
☒	☐ Animals and other organisms
☒	☐ Human research participants
☒	☐ Clinical data
☒	☐ Dual use research of concern

Methods

n/a	Involved in the study
☒	☐ ChIP-seq
☐	☒ Flow cytometry
☒	☐ MRI-based neuroimaging

Antibodies

Antibodies used

rabbit polyclonal anti-CTCF antibody (Cat.No.: #2899, Lot:2, Cell signaling Technology)
rabbit polyclonal anti-WAPL antibody (Cat.No. 16370-1-AP, Lot: 00052432, Proteintech)
mouse monoclonal anti-alpha tubulin antibody (Cat. No. #3873, Lot: 15, Cell Signaling Technology)
rabbit polyclonal anti-Rad21 antibody (Cat.No. ab154769, Lot: GR3224138-1, Abcam)

Validation

Anti-CTCF and anti-alpha-tubulin (CST): According to the manufacturer's website, both antibodies were validated for the use with mouse samples for Western Blotting. The manufacturer mentions the validation by SimpleChIP® Enzymatic Chromatin IP Kits for the anti-CTCF antibody. Anti-Rad21 (abcam): The manufacturer lists the antibody as being tested and suitable for the application in Western Blotting on mouse samples. Anti-WAPL (proteintech): The antibody was validated on samples from the same mouse cell lines in the following publication: Liu, N. Q. et al. "WAPL maintains a cohesin loading cycle to preserve cell-type-specific distal gene regulation." Nature Genetics 53, 100-109 (2021). Further validation is provided by this manuscript: The anti-CTCF, anti-Rad21 and anti-WAPL antibodies are validated by Western Blot in the CTCF/Rad21/WAPL-AID degron cell lines. In samples where the respective protein was degraded by IAA induction, no protein is detected, whereas in wild-type samples the protein is detected.

Eukaryotic cell lines

Policy information about cell lines

Cell line source(s)

All cell lines for the dual-array imaging are based on E14 mouse embryonic stem cells (mESCs) provided by Edith Heard laboratory, EMBL, Heidelberg. E14 CTCF-AID-eGFP (clone EN52.9.1) were published in Nora, Elphège P., et al. "Targeted degradation of CTCF decouples local insulation of chromosome domains from genomic compartmentalization." Cell 169.5 (2017): 930-944. E14 WAPL-AID-eGFP and E14 RAD21-AID-eGFP Liu, N. Q. et al. "WAPL maintains a cohesin loading cycle to preserve cell-type-specific distal gene regulation." Nature Genetics 53, 100-109 (2021). E14 WAPL-AID-eGFP were provided by Elzo de Wit laboratory, NKI, Amsterdam. E14 CTCF-AID-eGFP and E14 Rad21-AID-eGFP were provided by laboratory of Elphège Nora, University of California, San Francisco.

Authentication

Cell lines have been recurrently used by the authors in previous studies and therefore have not been authenticated.

Mycoplasma contamination

Cells were tested for mycoplasma contamination regularly and no contamination was detected.

Commonly misidentified lines
(See ICLAC register)

No commonly misidentified lines were used.

Flow Cytometry

Plots

Confirm that:

☒ The axis labels state the marker and fluorochrome used (e.g. CD4-FITC).

☒ The axis scales are clearly visible. Include numbers along axes only for bottom left plot of group (a 'group' is an analysis of identical markers).

☒ All plots are contour plots with outliers or pseudocolor plots.

☒ A numerical value for number of cells or percentage (with statistics) is provided.

Methodology

Sample preparation

Cells were treated with either 500 µM auxin or 500 nM dTag-13 for the indicated time and then harvested with Accutase and re-suspend in 1x PBS. Cells were then fixed in 4% paraformaldehyde for 15 min at RT and stained with 5 µg/ml DAPI for 30 min at RT.

Instrument

BD LSRII SORP Analyser (Becton Dickinson)

Software

BD FACSDiva™ Software v8.0.1, FlowJo (v10, BD Biosciences)

Cell population abundance	For each condition >50,000 cells were acquired.
Gating strategy	Forward scatter/Side scatter to discard big cells with high granularity; DAPI amplitude/DAPI height to discard doublets; DAPI amplitude/histogram to quantify cell cycle stage.

☒ Tick this box to confirm that a figure exemplifying the gating strategy is provided in the Supplementary Information.
