# Supplementary figures and images for: Cohesin and CTCF control the dynamics of chromosome folding
Source: Nat Genet. 2022 Dec 5;54(12):1907–18. doi: 10.1038/s41588-022-01232-7 (PMC9729113; doi:10.1038/s41588-022-01232-7)

## Source Data - Extended Data Figure 1A

Rad21:

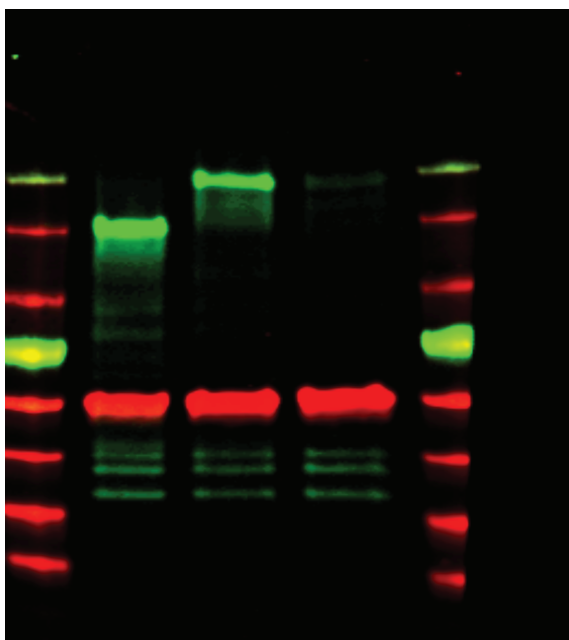

WAPL:

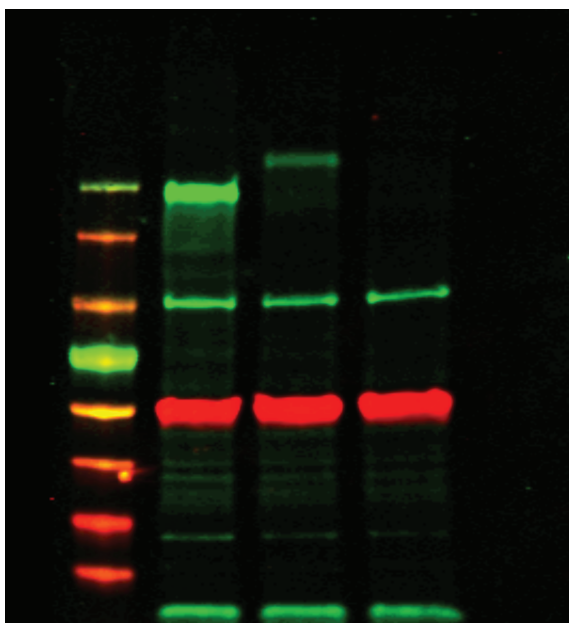

CTCF:

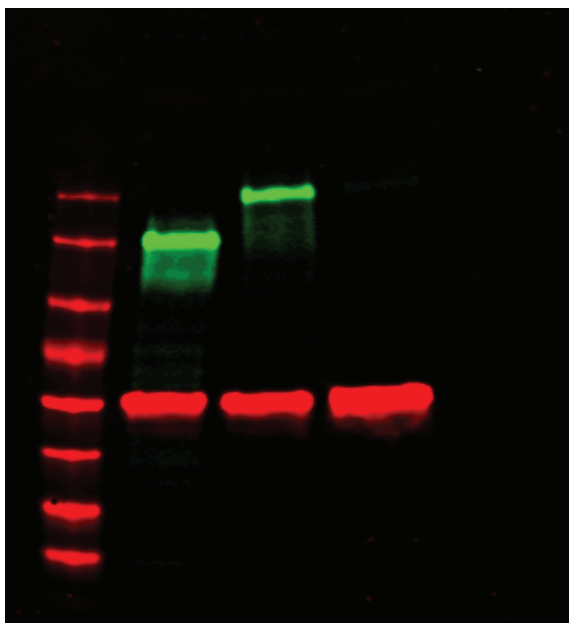

Supplement: Source Data Extended Data Fig. 1 — Unprocessed western blots and agarose gels. [file 41588_2022_1232_MOESM15_ESM.pdf]

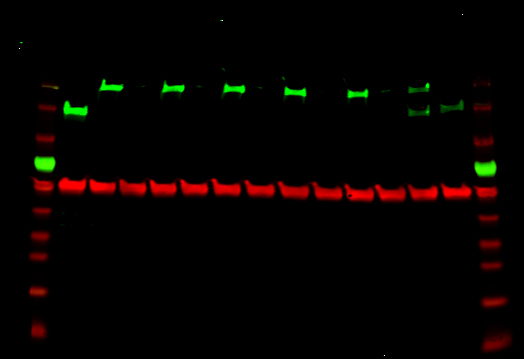

Supplement: Source Data Extended Data Fig. 5 — Unprocessed western blots. [file 41588_2022_1232_MOESM19_ESM.tif]
